# Raphe and ventrolateral medulla proteomics in epilepsy and sudden unexpected death in epilepsy

**DOI:** 10.1093/braincomms/fcac186

**Published:** 2022-07-12

**Authors:** Dominique F Leitner, Evgeny Kanshin, Manor Askenazi, Arline Faustin, Daniel Friedman, Sasha Devore, Beatrix Ueberheide, Thomas Wisniewski, Orrin Devinsky

**Affiliations:** Comprehensive Epilepsy Center, Grossman School of Medicine, New York University, 223 East 34th Street, New York, NY 10016, USA; Proteomics Laboratory, Division of Advanced Research Technologies, Grossman School of Medicine, New York University, 223 East 34th Street, New York, NY 10016, USA; Biomedical Hosting LLC, Arlington, MA 02140, USA; Department of Biochemistry and Molecular Pharmacology, Grossman School of Medicine, New York University, 223 East 34th Street, New York, NY 10016, USA; Center for Cognitive Neurology, Department of Neurology, Grossman School of Medicine, New York University, 223 East 34th Street, New York, NY 10016, USA; Department of Pathology, Grossman School of Medicine, New York University, 223 East 34th Street, New York, NY 10016, USA; Comprehensive Epilepsy Center, Grossman School of Medicine, New York University, 223 East 34th Street, New York, NY 10016, USA; Comprehensive Epilepsy Center, Grossman School of Medicine, New York University, 223 East 34th Street, New York, NY 10016, USA; Proteomics Laboratory, Division of Advanced Research Technologies, Grossman School of Medicine, New York University, 223 East 34th Street, New York, NY 10016, USA; Department of Biochemistry and Molecular Pharmacology, Grossman School of Medicine, New York University, 223 East 34th Street, New York, NY 10016, USA; Center for Cognitive Neurology, Department of Neurology, Grossman School of Medicine, New York University, 223 East 34th Street, New York, NY 10016, USA; Center for Cognitive Neurology, Department of Neurology, Grossman School of Medicine, New York University, 223 East 34th Street, New York, NY 10016, USA; Department of Pathology, Grossman School of Medicine, New York University, 223 East 34th Street, New York, NY 10016, USA; Department of Psychiatry, Grossman School of Medicine, New York University, 223 East 34th Street, New York, NY 10016, USA; Comprehensive Epilepsy Center, Grossman School of Medicine, New York University, 223 East 34th Street, New York, NY 10016, USA

**Keywords:** SUDEP, epilepsy, seizures, proteomics, brainstem

## Abstract

Brainstem nuclei dysfunction is implicated in sudden unexpected death in epilepsy. In animal models, deficient serotonergic activity is associated with seizure-induced respiratory arrest. In humans, glia are decreased in the ventrolateral medullary pre-Botzinger complex that modulate respiratory rhythm, as well as in the medial medullary raphe that modulate respiration and arousal. Finally, sudden unexpected death in epilepsy cases have decreased midbrain volume. To understand the potential role of brainstem nuclei in sudden unexpected death in epilepsy, we evaluated molecular signalling pathways using localized proteomics in microdissected midbrain dorsal raphe and medial medullary raphe serotonergic nuclei, as well as the ventrolateral medulla in brain tissue from epilepsy patients who died of sudden unexpected death in epilepsy and other causes in diverse epilepsy syndromes and non-epilepsy control cases (*n* = 15–16 cases per group/region). Compared with the dorsal raphe of non-epilepsy controls, we identified 89 proteins in non-sudden unexpected death in epilepsy and 219 proteins in sudden unexpected death in epilepsy that were differentially expressed. These proteins were associated with inhibition of EIF2 signalling (*P*-value of overlap = 1.29 × 10^−8^, *z* = −2.00) in non-sudden unexpected death in epilepsy. In sudden unexpected death in epilepsy, there were 10 activated pathways (top pathway: gluconeogenesis I, *P*-value of overlap = 3.02 × 10^−6^, *z* = 2.24) and 1 inhibited pathway (fatty acid beta-oxidation, *P*-value of overlap = 2.69 × 10^−4^, *z* = −2.00). Comparing sudden unexpected death in epilepsy and non-sudden unexpected death in epilepsy, 10 proteins were differentially expressed, but there were no associated signalling pathways. In both medullary regions, few proteins showed significant differences in pairwise comparisons. We identified altered proteins in the raphe and ventrolateral medulla of epilepsy patients, including some differentially expressed in sudden unexpected death in epilepsy cases. Altered signalling pathways in the dorsal raphe of sudden unexpected death in epilepsy indicate a shift in cellular energy production and activation of G-protein signalling, inflammatory response, stress response and neuronal migration/outgrowth. Future studies should assess the brain proteome in relation to additional clinical variables (e.g. recent tonic–clonic seizures) and in more of the reciprocally connected cortical and subcortical regions to better understand the pathophysiology of epilepsy and sudden unexpected death in epilepsy.

## Teaser

Sudden unexpected death in epilepsy occurs in 1 in 1000 epilepsy patients annually, but pathogenesis is poorly understood. Proteomic evaluation of implicated brainstem autonomic nuclei show differences in the midbrain dorsal raphe and fewer differences in the medullary raphe and ventrolateral medullary regions.

## Graphical Abstract

**Graphical Abstract:**
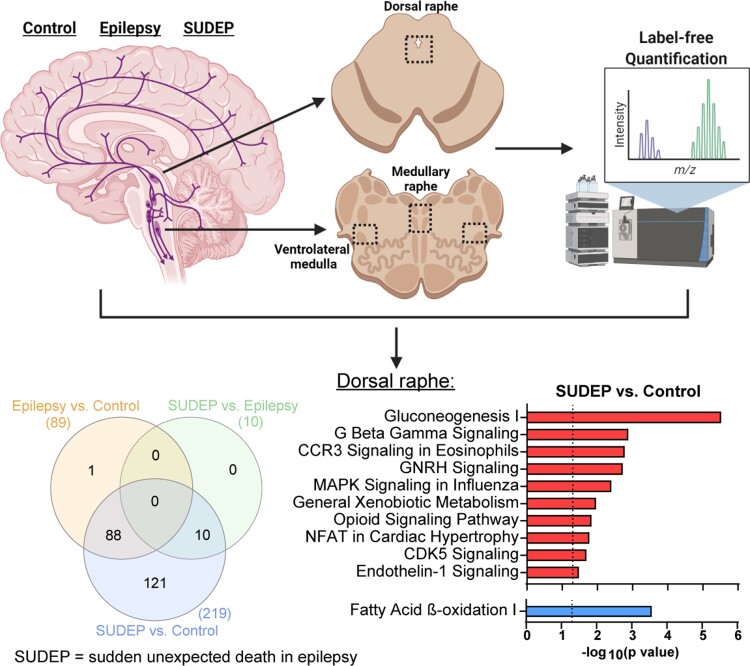

## Introduction

Sudden unexpected death in epilepsy (SUDEP) occurs in 1 in 1000 epilepsy patients every year, at higher rates in patients with treatment-resistant epilepsy.^1^ In epilepsy monitoring units, respiratory dysfunction often precedes SUDEP^2^ and likely results from brainstem dysfunction affecting autonomic and arousal systems.^1^ Prolonged postictal EEG suppression (PGES) after a generalized tonic–clonic seizure (GTCS) may impair arousal and respiration, causing a coma-like state with postictal central apnoea^3–5^ and hypoxia.^6,7^ Furthermore, the brainstem of SUDEP cases show medullary, pontine and midbrain atrophy.^8^ Compared with epilepsy patients who died from other causes and controls, SUDEP cases had decreased glia in medullary subregions.^9^ No molecular signature distinguishes SUDEP and other epilepsy cases in the brainstem, hippocampus or other cortical regions.^10–15^

Several brainstem autonomic nuclei are implicated in SUDEP.^16^ The ventrolateral medullary (VLM) region, with lower vimentin+ glia in SUDEP,^9^ contains the pre-Botzinger complex that modulates respiratory rhythm and reciprocally connects to brainstem and forebrain regions.^11,17–20^ The medial medullary raphe (MR), with lower connexin 43+ glia in SUDEP,^9^ modulates respiration via chemoreceptors with reciprocal projections to the lower brainstem and spinal cord. The medullary to lower midbrain serotonergic raphe are implicated in SUDEP and sudden infant death syndrome.^21,22^ In a SUDEP animal model, optogenetic activation of midbrain dorsal raphe (DR) serotonergic neurons suppressed tonic seizures and respiratory arrest,^23^ and another study showed decreased PGES length.^24^ The DR plays a role in arousal response to hypercapnia, with reciprocal projections to multiple regions in the forebrain, hippocampus and brainstem.^18,25–27^ There have been no brainstem proteomics studies in SUDEP or epilepsy cases to date.

In this study, we evaluated the proteomic molecular signalling networks associated with SUDEP and non-SUDEP epilepsy in diverse epilepsy syndromes and seizure types from autopsy tissue in the DR, MR and VLM.

## Materials and methods

### Human brain tissue

Post-mortem brain tissue was collected with approval by the New York University (NYU) School of Medicine Institutional Review Board. Cases were obtained through the North American SUDEP Registry (NASR), National Institutes of Health NeuroBioBank and NYU Center for Biospecimen Research and Development. NASR began enrolling cases from NYU, multiple clinical and forensic collaborators in October 2011, with all cases having written informed consent provided by next of kin. Cause of death was classified into non-SUDEP and SUDEP categories (definite SUDEP, definite SUDEP plus, probable SUDEP).^28^ Clinical history was determined from interviews and medical records. After neuropathological examination (T.W., A.F.) of NASR cases, brain tissue was processed into formalin-fixed paraffin-embedded blocks. Cases were selected with archival time in formalin <3 years, containing brainstem region of interest as confirmed anatomically and histologically for brainstem nuclei [tryptophan hydroxylase 2-positive (TPH2+) in both raphe nuclei; neurokinin receptor 1-positive (NK1R+) in VLM]. Cases were age- and sex-matched for available brain tissue fitting our criteria. Group sizes were determined by number of cases with significant findings as reported,^29–32^ including our epilepsy study with similar methods.^33^ Possible SUDEP, near SUDEP, and cases with insufficient information on cause of death were excluded. Our NASR cases were enrolled between May 2016 and June 2019 and included non-epilepsy controls (*n* = 4), non-SUDEP epilepsy (*n* = 16) and SUDEP (*n* = 25) cases. Non-epilepsy control (*n* = 15) and non-SUDEP epilepsy (*n* = 6) cases were obtained from the National Institutes of Health NeuroBioBank. Non-epilepsy control cases were also obtained from NYU Center for Biospecimen Research and Development (*n* = 2). Non-SUDEP categories of cause of death in the people with epilepsy (PWE) and control groups included cardiac arrest/disease, overdose/intoxication, trauma, drowning, pulmonary embolism, pneumonia, septic shock and suicide; see Supplementary Tables 1–3. PWE and SUDEP cases included diverse epilepsy syndromes. From a total of 68 cases, 137 regions for analysis with 15–16 cases/group in each brain region of interest were studied: 23 cases had all three brainstem nuclei regions available (23 cases only have DR) and 45 cases in medulla have both regions (one case has only MR). Case histories are summarized in Table 1 and detailed in Supplementary Tables 1–3 from cases with known information. Some NASR cases were not under regular medical care and had limited medical records. An overview schematic is provided in the Graphical Abstract, created with BioRender.com.

**Table 1:** Case history summary

Group	Cases	Mean age at death (years)	Sex	Mean PMI (hours)	Mean brain weight (grams)
Midbrain DR					
Control	15	37.1 ± 12.6	3 F/12 M	18 ± 6	1468 ± 99
PWE	16	43.1 ± 12.5	9 F/7 M	29 ± 12	1352 ± 207
SUDEP	15	31.7 ± 11.6	5 F/10 M	41 ± 22	1382 ± 206
Medulla MR					
Control	15	39.3 ± 10.4	2 F/13 M	23 ± 15	1457 ± 89
PWE	16	36.9 ± 14.3	9 F/7 M	38 ± 29	1347 ± 125
SUDEP	15	28.3 ± 10.2	5 F/10 M	35 ± 22	1419 ± 151
Medulla VLM					
Control	15	39.3 ± 10.4	2 F/13 M	23 ± 15	1457 ± 89
PWE	15	35.0 ± 12.4	9 F/6 M	35 ± 28	1349 ± 129
SUDEP	15	28.3 ± 10.2	5 F/10 M	35 ± 22	1419 ± 151

PMI = post-mortem interval.

PMI (*n* = 39 midbrain, *n* = 41 medulla) and brain weight (*n* = 40 midbrain, *n* = 43 medulla) are from cases with known information. There were 23 cases with all brain regions available. Both medullary regions were available in 45 cases. There are a total of 68 cases for all regions (Supplementary Tables 1–3).

### Brainstem nuclei identification

**Figure 1:**
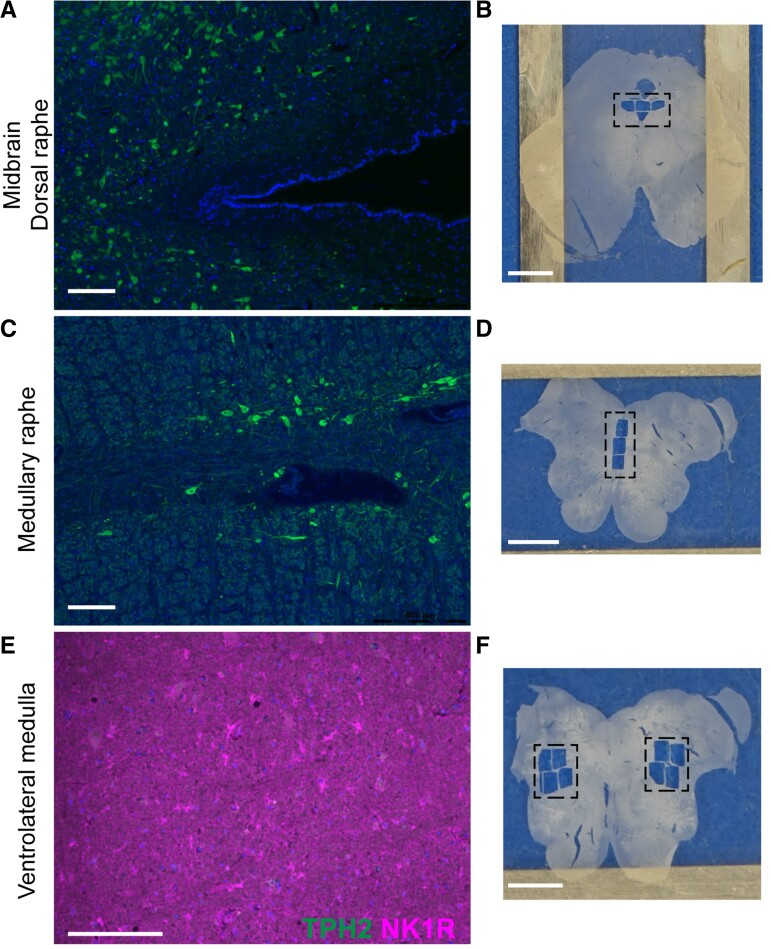
**Brainstem nuclei identification for LCM**. Immunohistological localization of brainstem nuclei confirmed brain tissue to be included for proteomics analyses on standard slides in a parallel section to the section on LCM slides that were subjected to the same immunohistology. (**A**) The midbrain DR were evaluated at the level of the inferior colliculus. The presence of the DR were confirmed by TPH2(+) neurons, depicted in a representative case. Scale bar represents 200 µm. (**B**) The region microdissected, 4.5 mm^2^, is depicted in the dashed box on an overview image on the right. Scale represents 5 mm. (**C**) The MR were evaluated in available brain tissue from one to four sections per case, from ∼6 to 10 mm above obex. The presence of the MR were confirmed by TPH2(+) neurons located medially, depicted in a representative case. Scale bar represents 200 µm. (**D**) The region microdissected, 4.5 mm^2^, is depicted in the dashed box on an overview image on the right. Scale represents 4 mm. (**E**) The VLM was evaluated in available brain tissue from 1 to 4 sections per case, from ∼6 to 10 mm above obex. The presence of the VLM was confirmed by NK1R(+) cells bilaterally with lateral localization, and TPH2(-) neurons, depicted in a representative case. Scale bar represents 200 µm. (**F**) The region microdissected, 12 mm^2^, is depicted in the dashed box on an overview image on the right. Scale bar represents 4 mm.

For all cases, midbrain was obtained at the inferior colliculus level and included the cerebellar decussation (caudal to the red nuclei) and medulla was isolated at 1 cm above obex and blocked into four levels within this 1 cm when available. To select nuclei of interest, all midbrain and medulla sections were subject to immunohistochemistry to confirm TPH2 in midbrain DR and medulla MR (containing both the raphe obscurus and raphe pallidus), as well as NK1R+/TPH2- in VLM on standard glass slides as described in the following context. The same histological markers were used to confirm nuclei localization on laser capture microdissection (LCM) slides as described.^34^ Representative photos were acquired with a SpotImaging camera using PathSuite 2.0 software (Fig. 1). NYU Center for Biospecimen Research and Development sectioned brain tissue for standard slides and NYU Experimental Pathology for LCM slides.

### Immunohistochemistry

To identify whether brainstem sections contained relevant nuclei, specific markers were evaluated before LCM. Briefly, 8 µm sections were deparaffinized and rehydrated through a series of xylenes and ethanol, followed by heat-induced antigen retrieval with 10 mM sodium citrate, 0.05% triton-x 100 at pH 6. After blocking in 10% normal donkey serum, sections were incubated overnight at 4°C with primary antibodies for TPH2 (1:250, Abcam ab121013) for midbrain or TPH2 and NK1R (1:100, Sigma S8305) for medulla. Secondary antibodies included donkey anti-goat Alexa-Fluor 488 (1:500, Thermofisher) and donkey anti-rabbit Alexa-Fluor 555 (1:500, Thermofisher), with nuclei counterstaining by Hoescht (1 mg/ml, Sigma B2261).

### Laser capture microdissection

To localize brainstem nuclei, 8 µm brain tissue sections on LCM compatible PET (polyethylene terephthalate) membrane slides (Leica) were immunostained with TPH2 (1:250, Abcam ab121013) for midbrain or TPH2 and NK1R (1:100, Sigma S8305) for medulla. Immunohistochemistry was performed as previously described with air drying overnight in a loosely closed container.^34^ For the VLM, an overview scan for the entire section before LCM allowed for NK1R+ and anatomical localization. Using LCM, we microdissected 12 mm^2^ of the VLM bilaterally and 4.5 mm^2^ for the DR and MR into mass spectrometry (MS) grade water (Thermo Scientific). Protein quantification from equal areas for each case allows for protein quantification with very low protein concentrations (estimated at <1 µg protein/sample). Microdissected samples were centrifuged for 2 min at 14 000 *g* and stored at −80°C. LCM was performed at 5 × magnification with a Leica LMD6500 microscope equipped with a UV laser.

Label-free Quantitative (LFQ) MS Proteomics: LFQ MS assessed differential protein expression, as described previously.^14,33^

Protein extraction and digestion were done according to the SPEED workflow.^35^ Microdissected samples were incubated in 10 µl of trifluoroacetic acid for 10 min at 70°C with subsequent quenching in 90 µl of 2 M Tris containing 10 mM tris(2-carboxyethyl)phosphine) and 40 mM 2-chloroacetamide. Samples were incubated at 95°C for 30 min, diluted with 500 µl of water containing 0.2 µg of sequencing grade trypsin (Promega). Digestions were performed overnight at 37°C and terminated by adding trifluoroacetic acid to final 2% (v/v). Peptides were loaded on C18 Evosep tips.

Peptides were separated using Evosep One LC system on 15 cm × 150 µm ID column packed with 1.9 µm ReproSil-Pur C18 beads (Evosep, cat# EV1113) over 44 min acetonitrile gradient (predefined by 30SPD Evosep Method) and analyzed on QExactive HF-X instrument (Thermo Scientific) in data-independent acquisition (DIA) mode doing MS2 fragmentation across 22 *m*/*z* windows after every MS1 scan event. High-resolution full MS spectra were acquired with a resolution of 120,000, an AGC target of 3e6, with a maximum ion injection time of 60 ms and scan range of 350–1650 *m*/*z*. Following each full MS scan, 22 data-independent HCD MS/MS scans were acquired at the resolution of 30,000, AGC target of 3e6, stepped NCE of 22.5, 25 and 27.5.

### Proteomics computational analysis

DIA MS data were analyzed in Spectronaut (https://biognosys.com/shop/spectronaut)^14,33^ and searched in directDIA mode against the SwissProt subset of the human Uniprot database (http://www.uniprot.org/). Database search was performed in integrated search engine Pulsar. For searching, the enzyme specificity was set to trypsin with the maximum number of missed cleavages set to 2. Oxidation of methionine was searched as variable modification; carbamidomethylation of cysteines was searched as a fixed modification. The false discovery rate (FDR) for peptide, protein and site identification was set to 1%. Protein quantification was performed on MS2 level using three most intense fragment ions per precursor. Data set was compared against 248 common laboratory contaminants^14,33^ and 28 proteins were removed. LFQ normalization was performed for each brain region separately.^36^ Subsequent data analysis and visualization were performed in the *R* environment for statistical computing and graphics (http://www.r-project.org/).

### Statistical analyses

The protein expression matrix (*n* = 2268) was filtered to contain only proteins that were quantified in ≥ 8 cases in at least one condition (control, PWE or SUDEP) in any brain region (*n* = 2237). For principal component analysis (PCA), missing values were imputed from the normal distribution with a width of 0.3 and downshift of 1.8 (relative to measured protein intensity distribution) in Perseus.^37^ A one-way ANOVA with *q* value correction followed by a Tukey’s *post hoc* test was performed to detect significant changes in protein expression among the control, PWE and SUDEP cases in the *R* environment (http://www.r-project.org/). Thresholds set for significance were an ANOVA *q* value < 0.05 calculated using the FDR method and a Tukey’s *post hoc P*-value < 0.05. A comparison of the proteins detected common to each region, as well as the significant proteins, were evaluated by Venn diagram generated from InteractiVenn.^38^ Cell-type-specific annotations were included in Supplementary Tables 4–6 and on volcano plots, from a reference data set^39^ and as described previously.^14,33^ Annotations were included when a protein had only one associated cell type and when the annotation included more than one associated cell type but were only neuronal proteins, for a total of 1066 possible annotations. Correlation analyses were performed by Pearson correlation in GraphPad Prism.

### Pathway analysis

The signalling pathways associated with the differentially expressed proteins in each region were assessed by Ingenuity Pathway Analysis (Qiagen). A core analysis was performed for each brain region with a threshold for each protein with ANOVA *q* value < 0.05 and Tukey’s *post hoc P*-value < 0.05. Coronavirus Pathogenesis Pathway is included in the supplemental table output but not included in the total number of significantly associated pathways as this study was performed before the COVID-19 pandemic.

### Data availability

All data needed to evaluate our conclusions are present in the article and Supplementary data. Additional data related to this article may be requested from the authors. MS RAW files were uploaded on MassIVE repository (https://massive.ucsd.edu/) with the following dataset ID MSV000088563.

## Results

### Brainstem nuclei identification

We confirmed midbrain DR by serotonergic-positive (TPH2+) neurons (Fig. 1A). The MR were identified by TPH2+ neurons, located medially 6–10 mm above obex (Fig. 1C). Localization of the VLM was confirmed by NK1R+ cells and TPH2- neurons laterally 6–10 mm above obex (Fig. 1E). The same histological markers were then used on LCM sections to guide microdissection in addition to anatomical landmarks.

### Proteomics differential expression analysis

**Figure 2:**
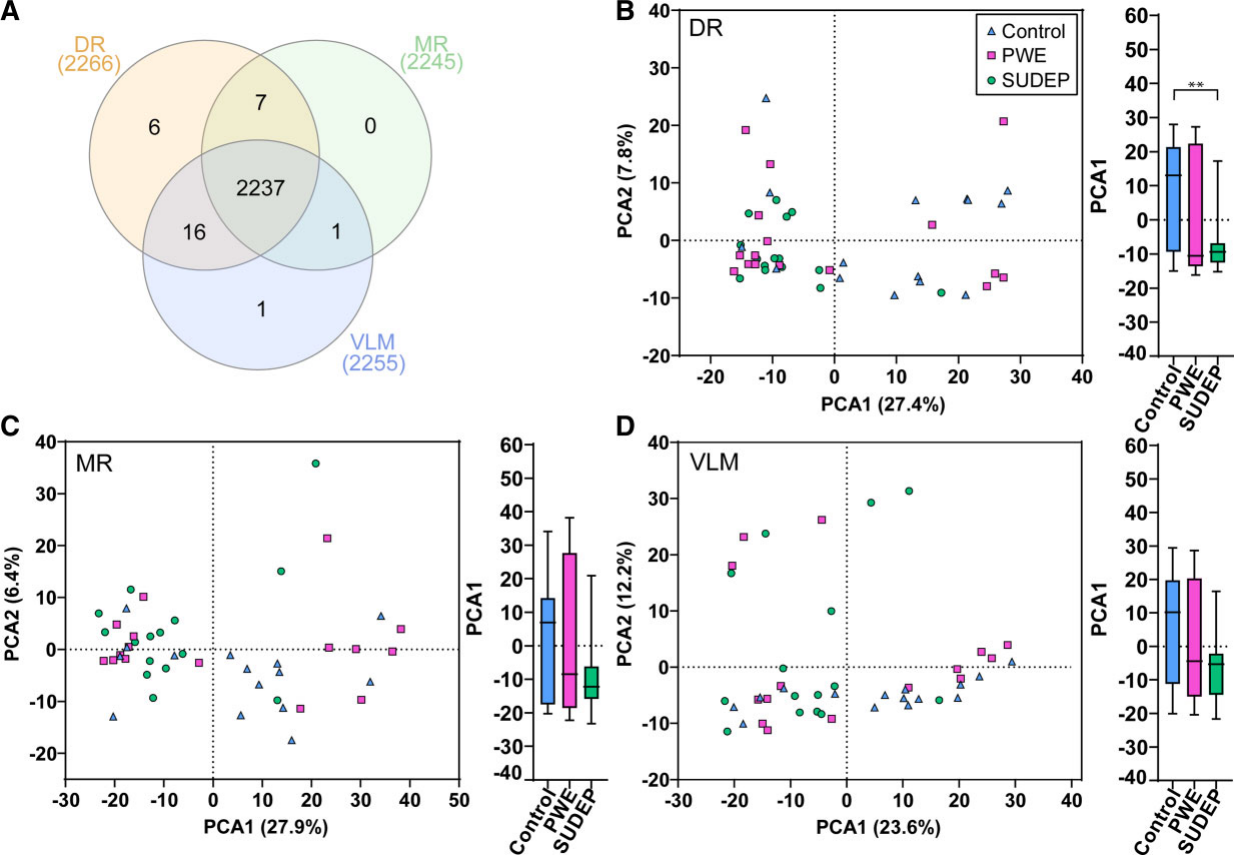
**Regionally detected proteins and PCA in the brainstem**. (**A**) Number of proteins detected in each of the brainstem regions analyzed, as well as overlap seen among all regions (*n* = 2237 proteins). In the DR, there were a total of 2266 proteins detected, with six unique to this region. In the MR, there were a total of 2245 proteins detected, with 0 unique to this region. In the VLM, there were a total of 2255 proteins detected, with one unique to this region. (**B**) PCA in the DR of all three groups, control, PWE and SUDEP cases. There is significant segregation of the SUDEP cases from the control cases in PCA1 (one-way ANOVA followed by Tukey’s *post hoc* test, *P* = 0.0083). (**C**) PCA in the MR indicates no significant segregation of the groups. (**D**) PCA in the VLM indicates no significant segregation of the groups.

From the three microdissected regions, 2268 proteins were detected in  ≥ 8 cases/group of a region with 2237 detected in all three regions (Fig. 2A). A PCA in each brain region indicated a significant separation in PCA1 of the DR between SUDEP and control cases (one-way ANOVA Tukey *post hoc P* = 0.0083, Fig. 2B). There was more variability in PCA1 for control and PWE cases in each brain region (Fig. 2B–D).

**Figure 3:**
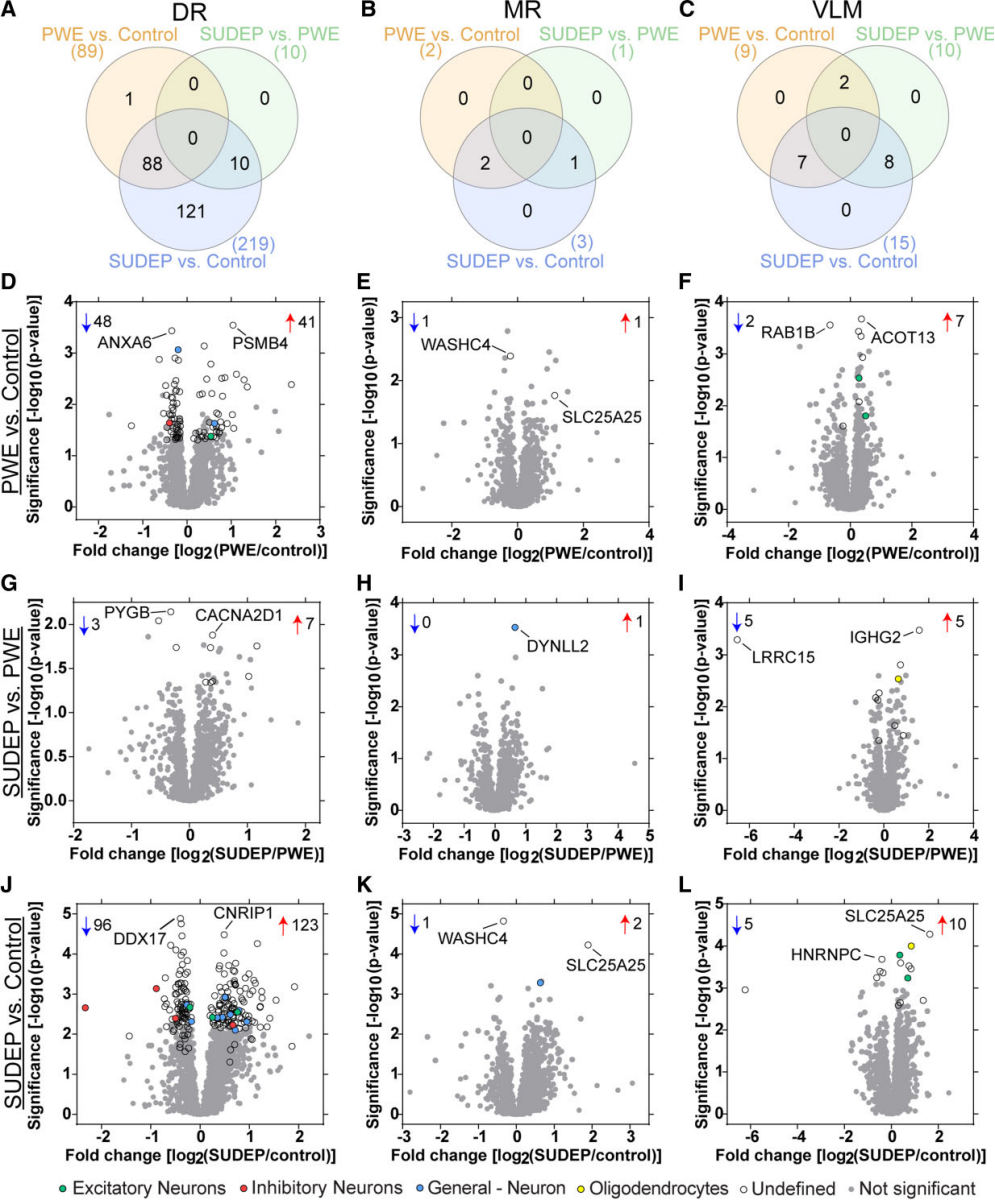
**Differential expression of proteins in the brainstem**. (**A**–**C**) Significantly altered proteins in each of the brainstem regions analyzed, as well as overlap seen in the pairwise comparisons. (**D**–**L**) Significantly altered proteins in each pairwise comparison are indicated after one-way ANOVA with *q* value correction (*q* < 0.05) followed by a Tukey’s post hoc test (*P* < 0.05, *y*-axis). The number of significantly increased (up arrow) and decreased (down arrow) proteins are indicated. The most significant protein that was increased and decreased are noted by gene name. Cell type annotation is also indicated for all significant proteins, detailed in the legend at the bottom. (**D**–**F**) PWE versus control differences are depicted for each of the brain regions, DR, MR and VLM, respectively. (**G**–**I**) SUDEP versus PWE differences are depicted for each brain region. (**J**–**L**) SUDEP versus control differences are depicted for each brain region.

Differential expression analysis using a one-way ANOVA with *q* value correction followed by a Tukey’s *post hoc* test, the largest protein changes were in the DR of SUDEP compared with control cases (219 proteins) followed by PWE compared with control cases (89 proteins; Fig. 3, Supplementary Table 4). Of changes in SUDEP cases, 131/219 proteins were uniquely significant to SUDEP and 10/219 proteins significantly differed between SUDEP and PWE (Fig. 3A). The top significant proteins identified in DR are summarized in Tables 2 and 3 and detailed in Supplementary Tables 4 and 7. Fewer protein differences were identified in the two medullary nuclei among all group comparisons (Fig. 3, Supplementary Tables 5, 6 and 8).

**Table 2:** Top 20 differentially expressed proteins in dorsal raphe of SUDEP versus control

Gene	Protein	UniProt ID	ANOVA *P*-value	ANOVA *q* value	Tukey *P*-value	Fold change
Decreased						
DDX17	Probable ATP-dependent RNA helicase DDX17	Q92841	2.18E-05	8.82E-03	1.29E-05	1.32
RPS3	40S ribosomal protein S3	P23396	2.60E-05	8.82E-03	1.76E-05	1.30
DDX3X	ATP-dependent RNA helicase DDX3X	O00571	4.24E-05	9.58E-03	3.54E-05	1.29
PLPP3	Phospholipid phosphatase 3	O14495	9.78E-05	1.54E-02	6.04E-05	1.51
HSD17B4	Peroxisomal multifunctional enzyme type 2	P51659	1.02E-04	1.54E-02	7.91E-05	1.40
FLII	Protein flightless-1 homologue	Q13045	6.13E-05	1.19E-02	8.93E-05	1.26
ANXA6	Annexin A6	P08133	3.96E-05	9.58E-03	1.08E-04	1.30
EEF2	Elongation factor 2	P13639	2.27E-04	2.20E-02	1.46E-04	1.20
EIF4A1	Eukaryotic initiation factor 4A-I	P60842	1.86E-04	2.10E-02	1.83E-04	1.28
ACAD9	Complex I assembly factor ACAD9, mitochondrial	Q9H845	4.07E-04	2.41E-02	2.58E-04	1.32
PFKM	ATP-dependent 6-phosphofructokinase, muscle type	P08237	3.82E-04	2.41E-02	2.83E-04	1.20
Increased						
CNRIP1	CB1 cannabinoid receptor-interacting protein 1	Q96F85	2.37E-05	8.82E-03	3.30E-05	1.40
PSMB4	Proteasome subunit beta type-4	P28070	2.01E-05	8.82E-03	5.50E-05	2.24
APP	Amyloid-beta precursor protein	P05067	1.60E-04	2.10E-02	9.42E-05	1.40
PSAT1	Phosphoserine aminotransferase	Q9Y617	2.80E-04	2.41E-02	1.69E-04	1.65
DDT	D-dopachrome decarboxylase	P30046	3.20E-04	2.41E-02	1.92E-04	1.50
SLC9A3R1	Na(+)/H(+) exchange regulatory cofactor NHE-RF1	O14745	2.07E-04	2.16E-02	1.97E-04	1.32
RAP2B	Ras-related protein Rap-2b	P61225	3.80E-04	2.41E-02	2.34E-04	1.30
LTA4H	Leukotriene A-4 hydrolase	P09960	4.18E-04	2.41E-02	2.59E-04	1.60
PGM1	Phosphoglucomutase-1	P36871	4.26E-04	2.41E-02	2.71E-04	1.63

**Table 3:** Top 20 differentially expressed proteins in dorsal raphe of PWE versus control

Gene	Protein	UniProt ID	ANOVA *P*-value	ANOVA *q* value	Tukey *P*-value	Fold change
Increased						
PSMB4	Proteasome subunit beta type-4	P28070	2.01E-05	8.82E-03	2.86E-04	2.05
CNRIP1	CB1 cannabinoid receptor-interacting protein 1	Q96F85	2.37E-05	8.82E-03	7.30E-04	1.31
PRPH	Peripherin	P41219	4.04E-04	2.41E-02	1.64E-03	1.45
NAXE	NAD(P)H-hydrate epimerase	Q8NCW5	4.16E-04	2.41E-02	2.60E-03	2.17
PTGR2	Prostaglandin reductase 2	Q8N8N7	2.66E-03	3.61E-02	3.02E-03	1.81
GSS	Glutathione synthetase	P48637	2.88E-03	3.63E-02	3.11E-03	1.72
RCN1	Reticulocalbin-1	Q15293	1.63E-03	3.12E-02	3.22E-03	1.36
CAST	Calpastatin	P20810	2.30E-03	3.39E-02	3.32E-03	2.44
CAPN5	Calpain-5	O15484	3.42E-03	3.63E-02	4.10E-03	5.12
SBSN	Suprabasin	Q6UWP8	6.22E-03	4.34E-02	4.58E-03	2.56
Decreased						
ANXA6	Annexin A6	P08133	3.96E-05	9.58E-03	3.68E-04	1.27
KIF21A	Kinesin-like protein KIF21A	Q7Z4S6	5.60E-04	2.62E-02	8.60E-04	1.15
FLII	Protein flightless-1 homologue	Q13045	6.13E-05	1.19E-02	1.25E-03	1.21
MX1	Interferon-induced GTP-binding protein Mx1	P20591	1.77E-04	2.10E-02	1.33E-03	1.55
ARPC2	Actin-related protein 2/3 complex subunit 2	O15144	6.92E-04	2.64E-02	1.38E-03	1.15
DDX3X	ATP-dependent RNA helicase DDX3X	O00571	4.24E-05	9.58E-03	3.44E-03	1.19
LYPLA2	Acyl-protein thioesterase 2	O95372	3.31E-03	3.63E-02	4.06E-03	1.22
TACO1	Translational activator of cytochrome c oxidase 1	Q9BSH4	3.58E-03	3.65E-02	4.32E-03	1.40
RPS3	40S ribosomal protein S3	P23396	2.60E-05	8.82E-03	5.29E-03	1.18
EIF4A1	Eukaryotic initiation factor 4A-I	P60842	1.86E-04	2.10E-02	5.59E-03	1.20

Most significant proteins have an ‘undefined’ cell type annotation, ubiquitously expressed in multiple cell types or the cell type association is unknown (Fig. 3, Supplementary Tables 4–6). For annotated proteins, most are neuronal or are specifically excitatory or inhibitory neuron. One significant protein was associated with a glial annotation; transferrin (Tf; P02787) was increased in the VLM of SUDEP cases compared to control (1.80-fold) and PWE (1.56-fold) cases.

**Figure 4:**
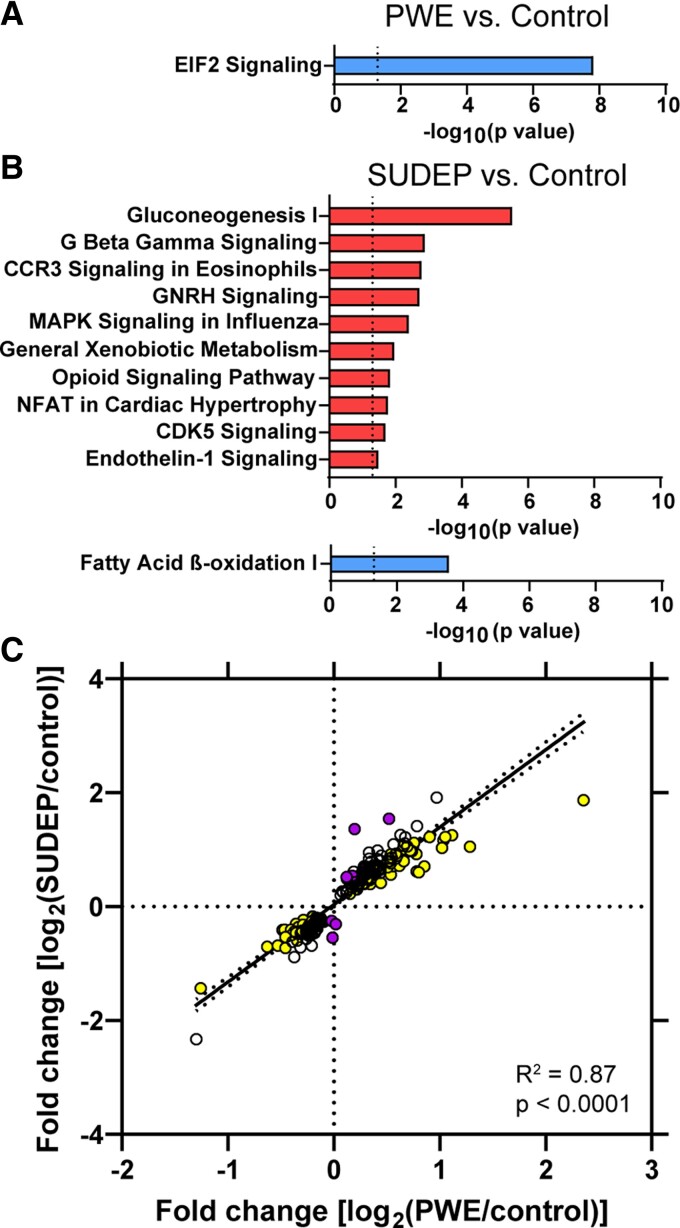
**Pathway analysis of differentially expressed proteins in the dorsal raphe**. (**A**) The 89 significant proteins in the DR of the PWE versus control comparison were significantly enriched for an inhibition of the EIF2 signalling pathway (*P*-value of overlap = 1.29 × 10^−8^, *z* = −2.00). (**B**) The 219 significant proteins in the DR of the SUDEP versus control comparison were significantly associated with ten activated pathways and one inhibited pathway, *P*-value of overlap and *z* scores detailed in Supplementary Table 10. (**C**) Of the 219 significant proteins in the DR of the SUDEP versus control comparison, proteins were trending in the same direction (up/down) in the PWE versus control comparison with a positive correlation (*P* < 0.0001, *R*^2^ = 0.87). Significance after ANOVA with *q* value correction followed by Tukey’s *post hoc* test is indicated by colour: yellow = PWE versus control and SUDEP versus control; white = SUDEP versus control; purple = SUDEP versus control and SUDEP versus PWE.

To determine the signalling pathways associated with protein changes, pathway analysis in the DR of PWE compared to control cases (89 proteins) indicated that these proteins were associated with 36 signalling pathways (*P*-value of overlap < 0.05) and one pathway was significantly impacted by fold change (|*z*| > 2, Supplementary Table 9). There was significant inhibition of EIF2 signalling (*P*-value of overlap = 1.28 × 10^−8^, *z* = −2.00, Fig. 4A). Comparing SUDEP and control cases in the DR (219 proteins), differentially expressed proteins were associated with 154 signalling pathways (*P*-value of overlap < 0.05), and there were 11 pathways significantly impacted by fold change (|*z*| > 2, Supplementary Table 10). There were 10 activated and 1 inhibited signalling pathways (Fig. 4B). The most significant activated pathway was gluconeogenesis I (*P*-value of overlap = 3.00 × 10^−6^, *z* = 2.24) and the inhibited pathway was fatty acid beta-oxidation I (*P*-value of overlap = 3.00 × 10^−6^, *z* = −2.00). EIF2 signalling was the most enriched pathway in PWE and SUDEP regardless of *z* score, reaching a significant *z* score in PWE while in SUDEP there were additional proteins differentially expressed resulting in a lower absolute *z* score for this pathway (*P*-value of overlap = 3.51 × 10^−13^, *z* = −1.67). Among 9 of the 11 significant signalling pathways in SUDEP versus control, there were shared proteins across these different signalling pathways related to G-protein signalling (e.g. increased CACNA2D1, CACNAD2, GNB2 and GNAS in G beta gamma, GNRH, opioid signalling). Among 4 of the 11 pathways, unique proteins were not shared with other signalling pathways: inflammatory response (CCR3 signalling in eosinophils, MAPK signalling in promoting pathogenesis of influenza), stress response (including PRDX6 in xenobiotic metabolism general signalling pathway, endothelin-1 signalling) and neuronal migration/outgrowth (CDK5 signalling). Comparing SUDEP and PWE in the DR, 19 pathways were associated with 10 altered proteins but none had a significant *z* score (|*z*| > 2, Supplementary Table 11).

In the medulla, few significant proteins were identified in the MR in all group comparisons; thus, no associated signalling pathways. In the VLM, there were fewer significant proteins identified with 60 pathways associated with the 9 proteins in PWE versus control, 37 pathways associated with the 15 proteins in SUDEP versus control and 7 pathways associated with the 10 proteins in SUDEP versus PWE (Supplementary Tables 12–14). None of the VLM pathways had a significant *z* score (|*z*| > 2).

Although no significant pathways overlapped between PWE and SUDEP when compared to control cases in the DR, the 219 significant proteins altered in SUDEP versus control cases were highly correlated to the fold changes seen in PWE versus control cases (*P* < 0.0001, *R*^2^ = 0.87, Fig. 4C). This indicates that many protein changes in SUDEP also trend in PWE versus control cases but do not reach significance.

**Figure 5:**
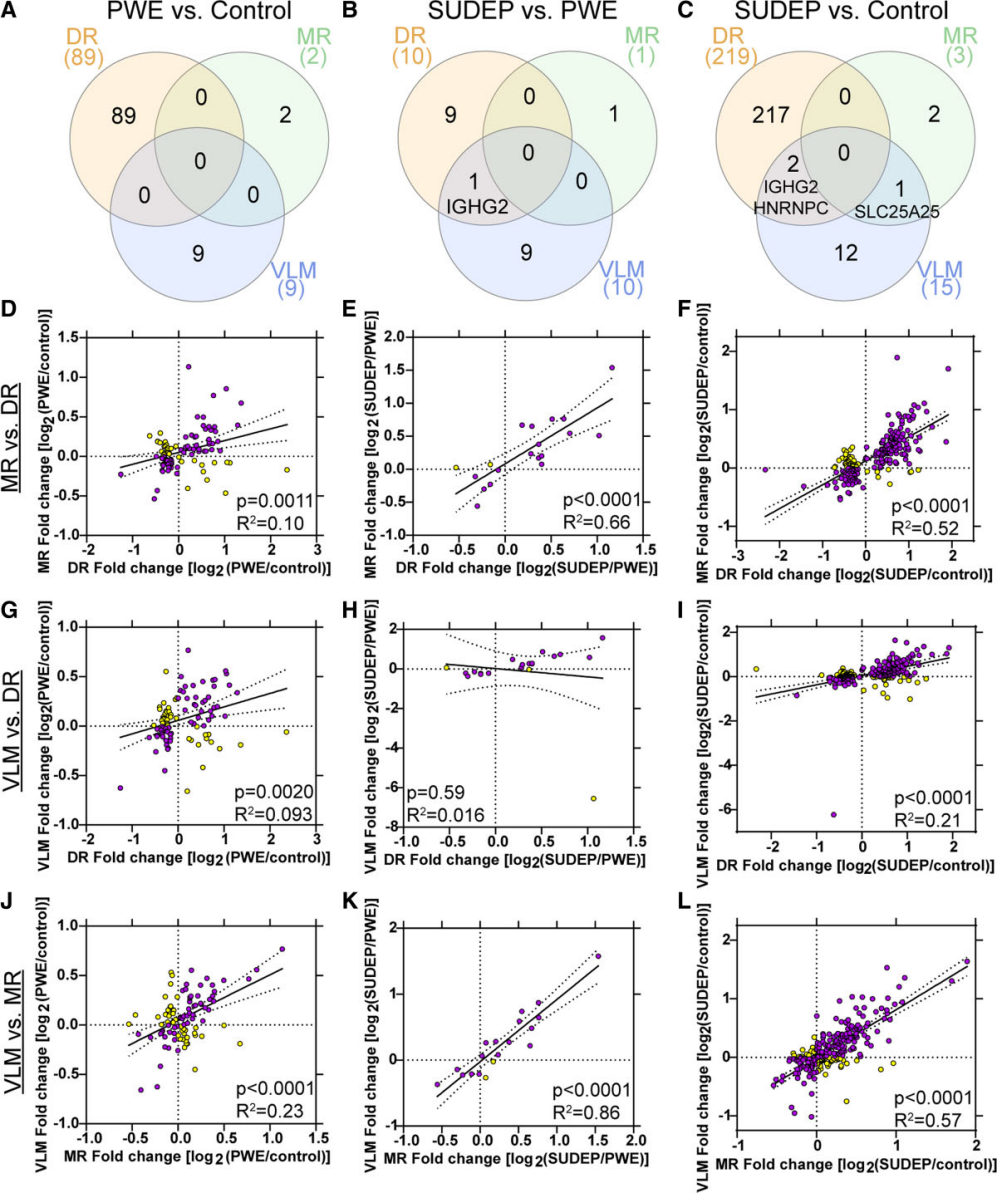
**Brainstem regional overlap of differentially expressed proteins**. Regional overlap of differentially expressed proteins after a one-way ANOVA with *q* value correction followed by a Tukey’s *post hoc* test. (**A**) When comparing PWE and control, there are no commonly significant proteins among the three regions analyzed. (**B**) When comparing SUDEP and PWE, there is one protein (IGHG2) that is increased in SUDEP in both the DR and VLM. (**C**) When comparing SUDEP and control, there are two proteins (IGHG2 increased, HNRNPC decreased) that are altered in both the DR and VLM. SLC25A25 is also increased in SUDEP in both the MR and VLM. (**D**–**L**) Correlation analyses between the various brainstem regions of all significant proteins, with included nuclei indicated on the left. (**D**, **G** and **J**) Correlation analyses indicated that the 100 significant proteins across all brainstem regions in PWE versus control were significant when comparing DR versus MR, DR versus VLM and MR versus VLM. (**E**, **H** and **K**) Correlation analyses indicated that the 20 significant proteins across all brainstem regions in SUDEP versus PWE were significant when comparing DR versus MR and MR versus VLM. There was no correlation in DR versus VLM, due to the difference in expression for LRRC15. This protein is detected in fewer cases in both of these regions, and it is not detected in the MR. (**F**, **I** and **L**) Correlation analyses indicated that the 234 significant proteins across all brainstem regions in SUDEP versus control were significant when comparing DR versus MR, DR versus VLM and MR versus VLM. In purple are the proteins with a fold change in the same direction and in yellow are proteins with a fold change in the opposite direction.

Regional overlap of significantly different proteins indicated no shared protein changes when comparing PWE versus control cases, three proteins when comparing SUDEP versus control cases and one protein when comparing SUDEP versus PWE cases (Fig. 5A–C). IGHG2 (Immunoglobulin Heavy Constant Gamma 2, P01859) was increased in the DR and VLM of SUDEP versus both PWE and control cases. In addition, in the DR and VLM, HNRNPC (Heterogeneous Nuclear Ribonucleoprotein C, P07910) was decreased in SUDEP when compared with control. In the MR and VLM, SLC25A25 (Solute Carrier Family 25 Member 25, Q6KCM7) was increased in SUDEP when compared to control cases. With few significant proteins having regional overlap, no signalling pathways were enriched. Correlation analyses indicated that of all significant protein changes in a region, there is a significant correlation with other brainstem regions analyzed except when comparing DR and VLM for SUDEP versus PWE due to a large difference in one protein (leucine rich repeat containing 15, LRRC15, Q8TF66, Fig. 5D–L). In the VLM, LRRC15 had a 93.58-fold change when comparing SUDEP and PWE (Supplementary Tables 6 and 8), which was largely due to the low detection of this protein in most cases indicated by both a lower LFQ value and with at least eight cases having detected protein in the PWE group but not in the control or SUDEP groups.

### Comparison to hippocampus and cortex

A comparison of the detected proteins in this data set to our previous non-SUDEP epilepsy data set in hippocampus and cortex^33^ (three overlapping cases) indicates 1976 common proteins to all brain regions, with 292 proteins unique to brainstem and 1240 proteins unique to the cortex and hippocampus (Supplementary Fig. 1A). The top signalling pathway associated with the unique brainstem proteins was coagulation system (*P*-value of overlap = 7.94 × 10^−12^) and in the hippocampus/cortex was synaptogenesis signalling pathway (*P*-value of overlap = 1.0 × 10^−20^). Of the shared proteins, the top enriched pathway was EIF2 signalling (*P*-value of overlap = 2.28 × 10^−54^). Of the significant proteins identified in the DR when comparing PWE versus control (*n* = 89 proteins), 81 were detected in cortex or hippocampus. There was a negative correlation of the fold change in proteins with the dentate gyrus (*P* = 0.0003, *R*^2^ = 0.17) and no correlation with the frontal cortex (*P* = 0.77, *R*^2^ = 0.0011) or hippocampus (*P* = 0.85, *R*^2^ = 0.00049; Supplementary Fig. 1B–D). EIF2 signalling was activated in the hippocampus (58 proteins) and frontal cortex (28 proteins), rather than inhibited as it was in the DR (10 proteins). There was a difference in the *P*-value of overlap; more significant in the hippocampus and cortex than the DR. Only two significant proteins were in the MR of PWE versus control (too few for a correlation analysis), which were detected in the cortex or hippocampal regions. In the VLM, seven of the nine significant proteins were detected in the cortex and hippocampal regions. There was a positive correlation in all regions: hippocampus (*P* = 0.063, *R*^2^ = 0.53), frontal cortex (*P* = 0.0069, *R*^2^ = 0.80) and in dentate gyrus (*P* = 0.0003, *R*^2^ = 0.94; Supplementary Fig. 1E–G). This indicates that significant protein changes in the VLM trend in the same direction of other brain regions when comparing PWE and control but not in DR.

Comparing detected proteins in this data set to our cortex and hippocampus SUDEP data set^14^ (three overlapping cases) indicates 1881 common proteins to all brain regions, with 387 proteins unique to brainstem and 966 proteins unique to cortex and hippocampus (Supplementary Fig. 1H). The top signalling pathway associated with the unique brainstem proteins was coagulation system (*P*-value of overlap = 1.41 × 10^−10^) and in hippocampus/cortex the synaptogenesis signalling pathway (*P*-value of overlap = 3.98 × 10^−21^). Of the shared proteins, the top enriched pathway was EIF2 signalling (*P*-value of overlap = 2.00 × 10^−52^). Of the significant proteins in the DR when comparing SUDEP versus PWE (*n* = 10 proteins), nine of these proteins were also detected in the cortex and hippocampus. There was a positive correlation with the hippocampus (*P* = 0.058, *R*^2^ = 0.42) and frontal cortex (*P* = 0.0036, *R*^2^ = 0.73), with a negative correlation in the dentate gyrus (*P* = 0.0086, *R*^2^ = 0.71; Supplementary Fig. 1I–K). There was only one significant protein (DYNLL2) in the MR of SUDEP versus PWE (too few for a correlation analysis), which was also detected in the cortex and hippocampal regions. In the VLM, 9 of the 10 significant proteins were detected in the cortex and hippocampal regions. There was a positive correlation in the hippocampus (*P* = 0.0020, *R*^2^ = 0.77) and frontal cortex (*P* = 0.0085, *R*^2^ = 0.71) but no clear correlation in the dentate gyrus (*P* = 0.80, *R*^2^ = 0.014; Supplementary Fig. 1L–N). This indicates that the significant protein changes in the brainstem trend in the same direction of other brain regions, except the dentate gyrus, when comparing SUDEP and PWE.

## Discussion

Our study identified altered proteins in the human raphe and VLM of the brainstem in diverse epilepsy syndromes that were more prominent in SUDEP cases, particularly in the midbrain DR. Top signalling pathways associated with these proteins indicated a shift in energy production (increased gluconeogenesis, decreased fatty acid beta-oxidation), and activated pathways associated with G-protein signalling, inflammatory response, stress response, and neuronal migration/outgrowth. DR changes in SUDEP also trended in PWE, supporting a potential progressive pathological process. A comparative analysis among brainstem regions and other previously evaluated cortical regions indicated similar global and region-specific protein changes in PWE and SUDEP.

### Midbrain

We identified changes in cellular energy production pathways in the midbrain DR of SUDEP, which may result from multiple pathogenic mechanisms or factors. Glucose metabolism may be impaired in epilepsy, which disrupts energy homeostasis that maintains neuronal membrane potentials and can foster seizure generation in a feed-forward cycle, demanding more energy to re-establish homeostasis and perform cellular repair.^40^ Impaired glucose metabolism may result from reduced glucose transport, decreased pyruvate dehydrogenase in oxidative metabolism and increased energy demands.^40^ PET imaging identified hypometabolism, reflecting low glucose transport and utilization, in epilepsy patients^41^ and in the frontal lobe of high-risk SUDEP patients.^42^ Uncontrolled seizures from anti-seizure medication (ASM) non-adherence, medication changes or withdrawal, and treatment-resistant epilepsy may disrupt homeostasis and increase energy needs. Many ASMs reduce neurotransmission, lowering brain energy requirements.^40^ Astrocytes regulate glycolysis and gluconeogenesis,^43^ providing an alternative glucose source via gluconeogenesis in ischaemic stroke and brain tumours (where lactate accumulates and inhibits glycolysis).^44^ In addition to altered gluconeogenesis in SUDEP, we found decreased fatty acid beta-oxidation, which normally occurs in astrocytes.^45^ The brain prefers glycolysis over fatty acid beta-oxidation, which requires less oxygen, minimizes superoxide production and oxidative stress response, and more rapidly generates ATP.^45^ Impaired astrocyte energy production in midbrain DR of SUDEP may reflect altered neuronal energy production or demands, astrocyte dysfunction, or a protective response to minimize oxidative damage in epilepsy.

The midbrain DR of SUDEP revealed activated pathways related to G-protein signalling (G beta gamma, GNRH, opioid signalling), inflammatory response (CCR3, MAPK signalling in promoting pathogenesis of influenza), stress response (xenobiotic metabolism general signalling pathway, endothelin-1 signalling), and neuronal migration/outgrowth (CDK5 signalling). We found a significant positive correlation of SUDEP proteins and PWE proteins in the midbrain DR (e.g. decreased EIF2 signalling), suggesting a progressive pathogenic process in neurons that mediate the arousal response to hypercapnia. MRI studies found brainstem atrophy from the medulla into the midbrain in SUDEP cases,^8^ activation of DR serotonergic neurons in an animal epilepsy model suppressed tonic seizures and respiratory arrest,^23^ and DR activation reduced PGES length.^24^ We did not detect changes related to serotonin signalling (TPH2 and SERT were not different, serotonin receptors were not detected). The midbrain of epilepsy patients is structurally normal.^15,46,47^ No proteomic or histological studies beyond neuropathology examined the midbrain in epilepsy patients. Our results and previous studies indicate that the role of the midbrain DR in SUDEP deserves further investigation, particularly on mechanism, astrocytes versus neurons, disease progression, ASMs and other factors.

### Medulla

In the medulla, we observed fewer protein changes in SUDEP and PWE. Previous studies of histological markers in medullary subregions identified differences between PWE or SUDEP to non-epilepsy controls (MBP, SYP, MAP2, GAL, SST, NK1R, TPH2, SERT).^11,13^ Vimentin+ and connexin 43+ astrocyte populations were decreased in SUDEP cases compared with PWE.^9^ In medullary subregions, no changes in markers of inflammation or blood–brain barrier (BBB) disruption (CD163, HLA-DR, IgG and albumin) were found in SUDEP cases.^10^ Few other medullary differences were identified in SUDEP versus PWE.^10–13,15^ Here, we observed one significant protein with a glial, oligodendrocyte, annotation (Tf). Tf was increased in the VLM of SUDEP compared with PWE and control cases (MBP, vimentin, connexin-43 were similar). In the brain, Tf is synthesized by oligodendrocytes and choroid plexus, and the protein is taken up by other cell types but may be present in capillary serum.^48^ Increased Tf in the highly myelinated VLM region may reflect processes related to myelin damage, increased cellular uptake of Tf, or BBB disruption. In epilepsy patients, decreased MBP occurs in medullary subregions,^13^ hippocampus and frontal cortex,^33^ as well as in the hippocampus of an epilepsy animal model.^49^ Increased Tf can occur after traumatic brain injury (TBI) or haemorrhage with BBB disruption.^50^ BBB disruption can occur in epilepsy, with IgG leakage, increased perivascular albumin and decreased ZO-1 in epilepsy patients and animal models.^51,52^ In our study, BBB permeability in the VLM was suggested by increased IGHG2 and HPX and decreased TJP1 (also known as ZO-1) in SUDEP compared with PWE and non-epilepsy controls. In addition, IGHG2 and HBG1 were increased in the DR of SUDEP compared with PWE. Changes to BBB permeability or vasculature changes in brainstem deserve further study, including whether these changes might be reflected in plasma or CSF as in TBI and aging.^53,54^ Future studies in the medulla may also be of interest to identify cell type- and nuclei- (i.e. raphe obscurus, raphe pallidus, arcuate nucleus) specific differences in SUDEP.

### Regional comparisons

A comparison of proteins across different brain regions indicates similar global and brain region-specific changes in PWE and SUDEP, with altered proteins in the DR being more unique. With few significant proteins identified in the medulla, there was little overlap of significant proteins among the brainstem regions analyzed. To determine whether there was a trend in similar proteins, an evaluation of significant proteins identified in at least one brainstem region of a pairwise comparison indicated a correlation across all brainstem regions (with the exception of one protein with low detection—LRRC15). Compared with our data^14,33^ in other cortical regions, there were fewer similarities but identified significant correlations of proteins in at least one brainstem region and cortical regions. Compared with PWE and controls, the significant VLM proteins trended in the same direction as other brain regions but not in DR (reflected by differences in activation/inhibition of the EIF2 signalling pathway). Comparing SUDEP and PWE, the significant brainstem protein changes trended in the same direction as other brain regions, except the dentate gyrus. We expected similar global- and region-specific protein changes, as in other studies.^55^ With reciprocally connected brain regions, it may be of interest in future studies to evaluate how disease duration influences these protein changes particularly with progressive midbrain atrophy observed in SUDEP by MRI^8^ and a negative correlation of brain weight and epilepsy duration in SUDEP.^15^

### Limitations

Our study had several limitations. Although we were able to detect regional differences in microdissected tissue, fewer large membrane proteins are detected by this technique. There was limited availability of brainstem tissue with specific regions of interest from all cases, thus not all cases had all three brain regions. Case referral to NASR was skewed by sources from the San Diego Medical Examiner Office (mainly low socio-economic white and Hispanic patients) and direct referrals (mainly high socio-economic white patients). We evaluated the broad changes evident from a heterogeneous group of cases with various epilepsy syndromes, seizure types and other clinical history. The availability of this critical brain region from well characterized cases is reinforced so that future studies can evaluate how the identified protein changes relate to specific clinical variables, i.e. epilepsy syndrome, seizure types, GTCS frequency and pathogenic gene variants.

### Conclusions

In summary, our study identified differential expression of proteins in the human epileptic raphe and VLM that were more pronounced in SUDEP, particularly in the midbrain DR. Top signalling pathways associated with these proteins indicated a shift in energy production, and increased G-protein signalling, inflammatory response, stress response, and neuronal migration/outgrowth. Future studies should evaluate how additional clinical variables from well characterized cases influence these protein changes (i.e. number of recent GTCS, ASMs), follow up mechanistic studies related to the proteomic signature identified in the DR, investigate specific epilepsy syndromes, and evaluate additional brainstem nuclei to understand and reduce SUDEP risk.

## Supplementary Material

fcac186_Supplementary_Data

## Abbreviations

ASM =: anti-seizure medication
BBB =: blood–brain barrier
COD =: cause of death
DIA =: data-independent acquisition
DR =: dorsal raphe
FDR =: false discovery rate
GTCS =: generalized tonic–clonic seizure
LCM =: laser capture microdissection
LFQ =: label-free quantification
MR =: medullary raphe
MS =: mass spectrometry
NA =: not applicable
NASR =: North American SUDEP Registry
ND =: not determined
NK1R =: neurokinin receptor 1
PCA =: principal component analysis
PET membrane =: polyethylene terephthalate membrane
PGES =: postictal generalized EEG suppression
PMI =: post-mortem interval
PWE =: people with epilepsy
SUDC =: sudden unexplained death in childhood
SUDEP =: sudden unexpected death in epilepsy
TBI =: traumatic brain injury
TPH2 =: tryptophan hydroxylase 2
VLM =: ventrolateral medulla
