# Supplementary material for: Raphe and ventrolateral medulla proteomics in epilepsy and sudden unexpected death in epilepsy
Source: Brain Commun. 2022 Jul 12;4(4):fcac186. doi: 10.1093/braincomms/fcac186 (PMC9344977; doi:10.1093/braincomms/fcac186)

**Supplementary Figure 1. Brainstem and previous cortical dataset correlations. A)** Overlap of detected brainstem proteins and previous dataset of PWE vs. control proteins. **B-G)** Correlation of significant proteins identified in brainstem (DR, VLM) and previous brain regions analyzed [hippocampal CA1-3 (HP), dentate gyrus (DG), frontal cortex (FC)] in PWE vs. control. There were too few proteins significant in the MR. Of those proteins significant in the DR for PWE vs. control, there were 81/89 detected in the brain regions analyzed in the previous dataset. There was a significant negative correlation in the DR and DG. Of those proteins significant in the VLM for PWE vs. control, there were 7/9 detected in the brain regions analyzed in the previous dataset. There was a significant positive correlation between the VLM and both the DG and FC. **H)** Overlap of detected brainstem proteins and previous dataset of SUDEP vs. PWE proteins. **I-N)** Correlation of significant proteins identified in brainstem (DR, VLM) and previous brain regions analyzed (HP, DG, FC) in SUDEP vs. PWE. There were too few proteins significant in the MR. Of those proteins significant in the DR for SUDEP vs. PWE, there were 9/10 detected in the brain regions analyzed in the previous dataset. There was a significant negative correlation for the DR and DG, and a significant positive correlation for the DR and FC. Of those proteins significant in the VLM for SUDEP vs. PWE, there were 9/10 detected in the brain regions analyzed in the previous dataset. There was a significant positive correlation between the VLM and both the HP and FC.


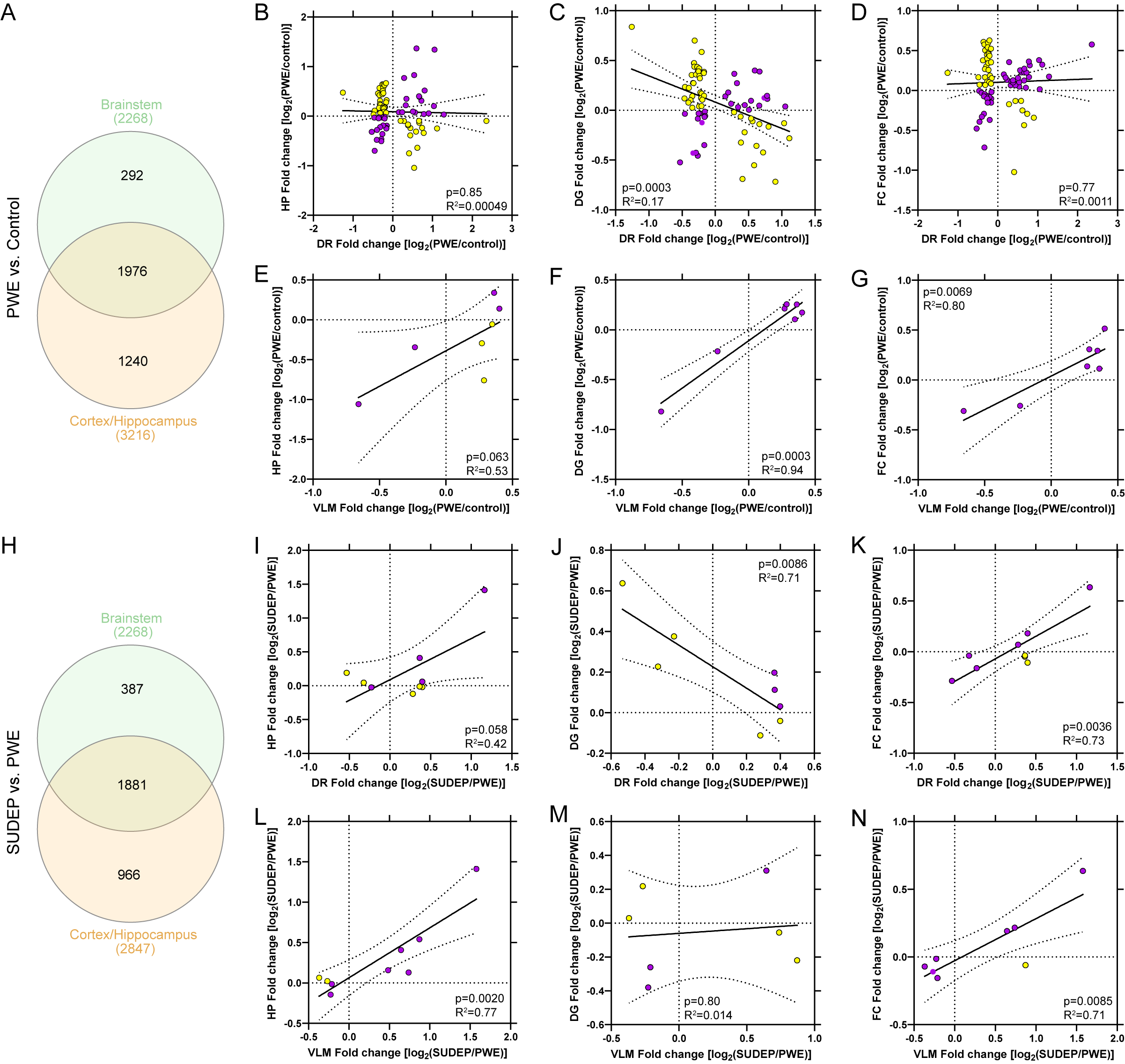

Supplement: fcac186_Supplementary_Data [file fcac186_supplementary_data.zip › Supplementary Figure 1.docx]
