# Supplementary material for: Raphe and ventrolateral medulla proteomics in epilepsy and sudden unexpected death in epilepsy
Source: Brain Commun. 2022 Jul 12;4(4):fcac186. doi: 10.1093/braincomms/fcac186 (PMC9344977; doi:10.1093/braincomms/fcac186)
Supplement: fcac186_Supplementary_Data [file fcac186_supplementary_data.zip › Supplementary Table Legends.docx]

**Supplementary Table Legends**

**Supplementary Table 1. Control, PWE, and SUDEP Case History in the Dorsal Raphe**

**Supplementary Table 2. Control, PWE, and SUDEP Case History in the Medullary Raphe**

**Supplementary Table 3. Control, PWE, and SUDEP Case History in the VLM**

**Supplementary Table 4. LC-MS/MS Data and ANOVA in the Dorsal Raphe**

**Supplementary Table 5. LC-MS/MS Data and ANOVA in the Medullary Raphe**

**Supplementary Table 6. LC-MS/MS Data and ANOVA in the VLM**

**Supplementary Table 7. All Differentially Expressed Proteins in Dorsal Raphe of SUDEP vs. PWE**

**Supplementary Table 8. All Differentially Expressed Proteins in Medulla of SUDEP vs. PWE**

**Supplementary Table 9. IPA Pathways in the Dorsal Raphe of PWE vs. Control**

**Supplementary Table 10. IPA Pathways in the Dorsal Raphe of SUDEP vs. Control**

**Supplementary Table 11. IPA Pathways in the Dorsal Raphe of SUDEP vs. PWE**

**Supplementary Table 12. IPA Pathways in the VLM of PWE vs. Control**

**Supplementary Table 13. IPA Pathways in the VLM of SUDEP vs. Control**

**Supplementary Table 14. IPA Pathways in the VLM of SUDEP vs. PWE**
